# Association Between Mandibular Cortical Erosion and Bone Mineral Density Assessed by Phalangeal Ultrasound and Dual Energy X-Ray Absorptiometry in Spanish Women

**DOI:** 10.3390/diagnostics15040507

**Published:** 2025-02-19

**Authors:** Maria L. Canal-Macías, Vicente Vera-Rodríguez, Olga Leal-Hernández, Julián Fernando Calderón-García, Raúl Roncero-Martín, Francisco García-Blázquez, Sergio Rico-Martín, Fidel López-Espuela, José M. Morán, Juan Fabregat-Fernández, Jesús M. Lavado-García, María Pedrera-Canal

**Affiliations:** 1 Metabolic Bone Diseases Research Group, Nursing Department, Nursing and Occupational Therapy College, University of Extremadura, 10003 Cáceres, Spain; luzcanal@unex.es (M.L.C.-M.); vicenteverarod@gmail.com (V.V.-R.); rronmar@unex.es (R.R.-M.); fidellopez@unex.es (F.L.-E.); jmmorang@unex.es (J.M.M.); juanfabregat@unex.es (J.F.-F.); jmlavado@unex.es (J.M.L.-G.); 2 Department of Nursing, Nursing and Occupational Therapy College, University of Extremadura, 10003 Cáceres, Spain; jfcalgar@unex.es (J.F.C.-G.); sergiorico@unex.es (S.R.-M.); 3 Department of Nursing, University Centre of Plasencia, University of Extremadura, 10600 Plasencia, Spain; pacoblaz@unex.es; 4 Nuclear Medicine Service, Hospital San Carlos Clinical, Servicio Madrileño de Salud, 28040 Madrid, Spain; maria.pedrera@salud.madrid.org

**Keywords:** osteoporosis, mandibular cortical erosion, bone mineral density, panoramic radiography, Klemetti index

## Abstract

**Background and Objectives**: Analysing the characteristics of the mandibular bone through panoramic radiographs could be useful as a prescreening tool for detecting individuals with osteoporosis. The aims of this study were to evaluate the possible associations between the mandibular cortical index (MCI) and bone mineral density (BMD) in various bone regions, to investigate whether BMD better identifies moderate–severe mandibular erosion or severe mandibular erosion, and to establish BMD cut-off points to identify individuals with moderate or severe mandibular cortical erosion. **Methods**: This study analysed 179 Spanish Caucasian women between September 2021 and June 2024. Bone measurements, including amplitude-dependent speed of sound (Ad-SOS), the ultrasound bone profiler index (UBPI), and the bone transmission time (BTT), were obtained via dual energy X-ray absorptiometry (DXA) for the femoral neck, lumbar spine, and trochanter and quantitative bone ultrasound (QUS) for the phalanx. The MCI was calculated via the Klemetti index from panoramic radiographs. **Results**: According to the Klemetti index classification, lower QUS measurements in the phalanx and DXA measurements in the femoral neck, trochanter, and lumbar spine were found in women with poorer mandibular cortical bone quality. Our results revealed that, compared with moderate cortical erosion, all the BMD measures had better AUCs when identifying severe cortical erosion. Moreover, femoral neck BMD had the largest area under the curve (AUC = 0.719) for detecting severe mandibular cortical erosion, suggesting a cut-off of <0.703 gr/cm^2^. Finally, predictor analysis of osteoporosis revealed that moderate and severe mandibular cortical erosion, compared with an uninjured mandibular cortical area, was independently associated with a diagnosis of osteoporosis. **Conclusions**: In conclusion, MCI was associated with BMD measurements assessed by QUS and DXA in various bone regions. Our results suggest that the Klemetti index could be used as a predictor of osteoporosis and fracture risk.

## 1. Introduction

Osteoporosis is a common disease characterized by a decrease in bone density and the breakdown of bone structure, which predominantly affects postmenopausal women, but also affects older men [1]. It is considered a skeletal disorder marked by reduced bone strength, which makes bones more susceptible to fractures [1,2]. Currently, osteoporosis is diagnosed through dual-energy X-ray absorptiometry (DXA), which measures the bone mineral density (BMD) for the femoral neck, total hip, lumbar spine, and/or the 1/3 radius [3,4]. The results are expressed as a T score using appropriate reference data according to age, sex, and ethnicity. However, DXA is not available worldwide, and its cost limits its use in many countries [5]. This limitation has prompted numerous researchers to look for more affordable and accessible alternatives to DXA, such as ultrasound (QUS), peripheral DXA or quantitative computer tomography (QCT), to diagnose osteoporosis or predict the risk of future fractures [6,7,8,9].

Studies have confirmed a possible association between skeletal bone mass and mandibular bone mass [10]. Panoramic radiographs are low-cost procedures utilized as diagnostics before dental treatment, and they have potential applications beyond the dental field. Researchers have explored the usefulness of dental radiographs in the early diagnosis of osteoporosis through the specialized use of panoramic images [10,11]. Several mandibular cortical indices, including the mandibular cortical index (MCI), gonial index (GI), antegonial index (AI), panoramic mandibular index (PMI), and mental index (MI), have been proposed to evaluate the quality of mandibular bone mass on the basis of panoramic radiograph images [12,13].

One of the most well-established methods for assessing bone quality by panoramic radiography is the qualitative evaluation of the MCI according to the Klemetti index [14]. This index was created to estimate the mandibular cortical bone in the mandibular foramen area. It has been suggested that in individuals with osteoporosis, the mandibular cortical bone undergoes resorption, which can be observed using dental panoramic radiographs. Most studies that have investigated the relationship between MCI and BMD have suggested that the Klemetti index may be helpful for detecting individuals with osteoporosis or low skeletal BMD [15]. However, there is still debate over whether to consider only severely eroded cortexes or include moderately eroded cortexes when screening for osteoporosis, as only a limited number of studies have assessed the diagnostic accuracy of severely eroded cortexes [10]. Moreover, only one study has investigated the association of MCI with the bone mineral status assessed by phalanx QUS [16]. In addition, no studies have been performed in the Spanish population, and no studies have established a BMD cut-off that is adequate for identifying individuals with moderate or severe mandibular cortical erosion.

The aims of this study were to evaluate the possible associations between MCI and BMD assessed by QUS and DXA in various bone regions, to investigate whether BMD measurements better identify moderate–severe mandibular erosion or severe mandibular erosion, and to establish BMD cut-off points to identify individuals with moderate or severe mandibular cortical erosion.

## 2. Methods

### 2.1. Study Sample

This cross-sectional study was carried out between September 2021 and June 2024. It included 179 Caucasian women who consulted the GIEMO (Grupo de Investigación en Enfermedades Metabólicas Óseas) research group of the Nursing and Occupational Therapy College of Cáceres, Spain (University of Extremadura). The sample size was calculated on the basis of the study by Leite et al. [17] to determine the minimum number of participants in each of the three groups according to the MCI classification. A minimum of 31 subjects were necessary to detect a medium–large effect size on the BMD of the femoral neck (Cohen’s f = 0.33, α = 0.05, and power = 80%). Each MCI group in this study included more than 31 participants. Women with mental or physical disabilities, terminal disease, bone fractures, pregnancy, dietary restrictions or anti-osteoporotic drug treatment were excluded from this study. Each subject participating in this study provided written informed consent, and the ethics committee of the University of Extremadura approved the study design (reference number: 192/2020) in December 2020.

### 2.2. Study Variables

Biological data such as age, age at menarche, gonadal status, years of menopause, pregnancies, births, and months of breastfeeding were collected from all participants. Postmenopausal status was defined when at least 12 months had passed since the last menstrual period. Calcium and/or vitamin D supplementation and smoking status data were also collected. Physical activity was evaluated using the International Physical Activity Questionnaire (IPAQ) [18].

Anthropometric parameters were measured according to the recommendations of the Spanish Society for the Study of Obesity [19]. Height, weight, waist circumference, and hip circumference were determined to calculate the body mass index (BMI) and waist-to-hip ratio (WHR). A Tanita BC-418 MA Segmental Body Composition Analyzer (Tanita Corp., Tokyo, Japan) was used to determine the amount of body fat.

### 2.3. Mandibular Cortical Assessment

A previously trained operator performed mandibular panoramic radiography on each participant via a Ratograph EVO 3D (Villa Sistemi Medicali, Milan, Italy) device at 72 kV, 6 mA, and 14.4 s exposure. The resulting images were saved in JPEG format with a resolution of 1536 × 2573 pixels and were assessed with the MATLAB R2018b software.

MCI status was classified by the Klemetti index [14] according to the mandibular foramen area: C1 (the endosteal margin of the cortex is even and sharp on both sides), C2 (the endosteal margin shows semilunar defects (lacunar resorption) and seems to form endosteal cortical residues on one or both sides) or C3 (the cortical layer becomes porous and exhibits heavy endosteal cortical residue).

### 2.4. Bone Mineral Density Assessments

QUS measurements were carried out by a trained operator on the 2° to the 5° proximal phalanx of the nondominant hand using a DBM Sonic Bone Profiler (IGEA, Capri, Italy). This device measures the amplitude-dependent speed of sound (Ad-SOS) in m/s, the ultrasound bone profiler index (UBPI), and the bone transmission time (BTT) in μs.

The trochanter BMD, L2–L4 BMD lumbar spine BMD, and femoral neck BMD measurements were carried out via DXA using a Norland XR-800 (Fort Atkinson, WI, USA) and expressed as the quantity of mineral divided by the area scanned (g/cm^2^). According to the World Health Organization (WHO) criteria [20], we diagnosed osteoporosis when patients obtained T scores < −2.5 in any of the areas assessed by DXA. The T score is defined as the standard deviation between a subject’s BMD and that of a young adult reference population.

### 2.5. Statistical Analysis

Statistical analyses were performed using the statistical package IBM SPSS version 28.0 (IBM Corporation, Armonk, NY, USA) for Windows. Categorical variables were expressed as frequencies (percentage) and compared with Pearson’s chi-square test. The normal distribution was confirmed by the Kolmogorov–Smirnov test. Quantitative variables were expressed as means ± standard deviations if they were normally distributed and as medians [interquartile ranges] otherwise. The participants were compared according to the Klemetti index stratification. Analysis of variance (ANOVA) was performed with the Bonferroni post hoc correction. Pairwise comparisons between subgroups were performed with the Kruskal–Wallis test and the Mann–Whitney U test for non-normally distributed data. Moreover, the average BMD values were compared between groups via analysis of covariance (ANCOVA), adjusting for age (years), gonadal status (pre- or postmenopausal), age at menarche (years), years of menopause (years), BMI (kg/m^2^), WHR, % body fat, Ca supplementation, vitamin supplementation, physical activity, and smoking.

The area under the curve (AUC) and the corresponding 95% confidence interval (CI) were estimated by receiver operating characteristic (ROC) analysis. The optimal BMD cut-off values for detecting moderate or severe mandibular cortical erosion (Klemetti index = C2 or C3) from ROC analyses were examined by the maximum Youden index (sensitivity + specificity − 1).

Finally, univariate and multivariate logistic regression analyses were performed to study the probable associations between the presence of osteoporosis (dependent variable) and the independent variable, in order to study whether the Klemetti index was independently associated with the presence of osteoporosis. Odds ratios (ORs) and corresponding 95% CIs were estimated. Adjusted ORs (aORs) were determined via multivariate analysis, where independent variables were included when *p* < 0.10 was found in the univariate analysis.

## 3. Results

**Figure 1:**
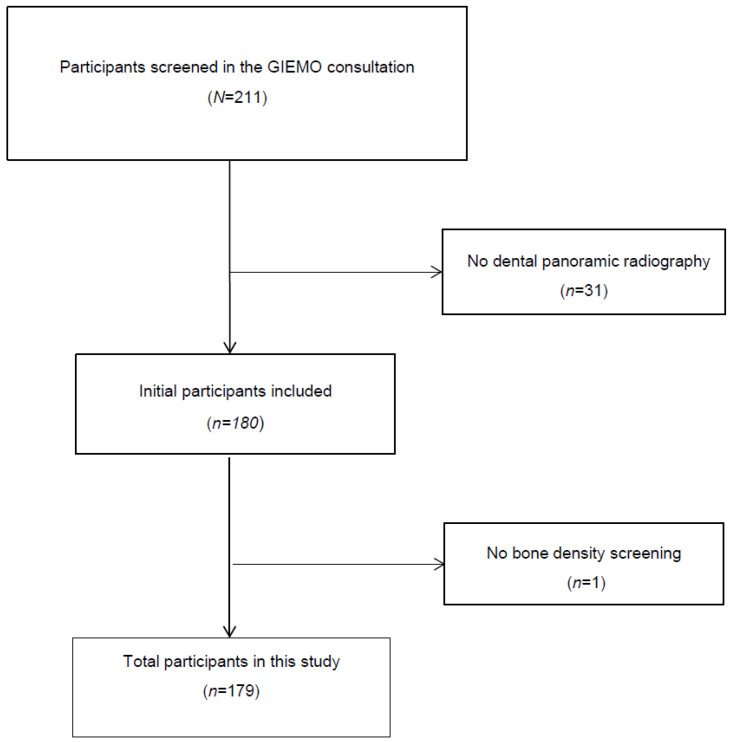
Participant selection process.

Initially, 211 women were screened, 31 of whom were excluded because no dental panoramic radiography assessment was available (Figure 1). A total of 180 women were recruited; however, 1 was excluded because BMD measurements could not be performed due to a hip prosthesis. Finally, 179 women (87.2% postmenopausal) with a mean age of 58.57 ± 9.44 years were investigated. Among these patients, 44 (24.6%) had osteoporosis diagnosed by DXA. The participants were classified according to the Klemetti index as C1 (26.8%), C2 (54.2%) or C3 (19.0%). The main baseline characteristics of the participants according to the Klemetti index are shown in Table 1. Significant differences were found between groups in terms of age, years of menopause, and tooth loss. Compared with those in group C1, women with a Klemetti index of C3 were significantly older and had significantly more years of menopause and more tooth loss.

Table 2 describes the relationships between Klemetti index groups and quantitative bone ultrasound and BMD assessment by DXA. We found statistically significant differences in all the bone density variables studied. Post hoc tests revealed significant differences between C1 and C2 in Ad-SOS and UBPI; between C2 and C3 in Ad-SOS, UBPI, femoral neck BMD, trochanter BMD, and lumbar spine BMD; and between C1 and C3 in each bone density variable analysed. After adjustments for age, gonadal status, age of menarche, years of menopause, BMI, WHR, % body fat, Ca supplementation, vitamin supplementation, physical activity, and smoking, significant differences were also found in all the bone density variables studied except for the BTT. Post hoc tests revealed significant differences between C1 and C2 for Ad-SOS and UBPI and between C1 and C2 for Ad-SOS, UBPI, femoral neck BMD, trochanter BMD, and lumbar spine BMD. In addition, significant differences were observed between the three groups according to the Klemetti index with regard to the diagnosis of osteoporosis. Compared with women in groups C2 and C1, women in group C3 had a greater proportion of osteoporosis, and women in group C2 had a significantly greater percentage of osteoporosis than women in group C1.

**Figure 2:**
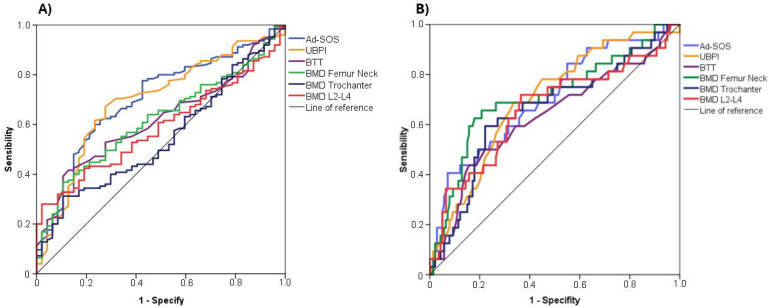
ROC analysis to predict moderate or severe cortical erosion (**A**) and severe cortical erosion (**B**).

According to the ROC analyses (Table 3 and Figure 2A) used to identify moderate or severe mandibular cortical erosion (Klemetti index = C2 or C3), the AUCs obtained for the BMD measures were inferior to the AUCs for quantitative bone ultrasound. Ad-SOS and UBPI had the largest areas under the curve (AUCs: 0.702 and 0.695, respectively), and the BMD trochanter had the smallest AUC (AUC: 0.543). The best AUC for the DXA measurement was observed for the femoral neck BMD (AUC 0.617). The optimal cut-off values to detect moderate mandibular cortical erosion from the ROC analyses were <2037.5 for Ad-SOS and <0.714 for femoral neck BMD. Furthermore, in the ROC analyses (Table 3 and Figure 2B) for detecting severe mandibular cortical erosion (Klemetti index = C3), femoral neck BMD and Ad-SOS had the largest areas under the curve (AUC: 0.719 and 0.703, respectively) and BTT had the smallest AUC (AUC: 0.632). Both quantitative bone ultrasound and BMD measurements achieved significant AUCs (*p* < 0.05). The optimal cut-off values for identifying mandibular severe cortical erosion from ROC analyses were <0.703 for femur neck BMD and <1929.00 for Ad-SOS.

Finally, univariate and multivariate logistic regression analyses were conducted to investigate the possible associations between the diagnosis of osteoporosis and independent variables (Table 4). In the univariate analyses, age, postmenopausal status, age at menarche, years of menopause, Ca and vitamin D supplementation, tooth loss, and a Klemetti index of C2 (OR: 2.82; 95% CI: 1.01–7.95; *p* = 0.045) and C3 (OR: 6.78; 95% CI: 2.15–21.38; *p* = 0.001) with respect to C1, presented a significant positive association with the presence of osteoporosis. However, in the multivariate analysis, only vitamin D supplementation (OR: 3.91; 95% CI: 1.01–15.19; *p* = 0.049) and a Klemetti index of C2 (OR: 3.70; 95% CI: 1.12–12.18; *p* = 0.031) or C3 (OR: 7.88; 95% CI: 2.03–30.52; *p* = 0.003) with respect to C1 were significantly associated with the diagnosis of osteoporosis.

## 4. Discussion

In this cross-sectional study, which included 179 women, MCI was associated with BMD measurements assessed by QUS and DXA in various bone regions.

Early identification of osteoporosis is crucial to enable the prevention of fractures. Currently, clinical practice guidelines consider DXA in the femoral neck and lumbar spine as the gold standard technique for the diagnosis of osteoporosis; however, access to screening is often limited in many countries [21,22,23]. Alternatively, other diagnostic techniques, such as QUS, peripheral DXA or QCT, have been proposed to diagnose osteoporosis and predict the risk of future fractures [6,7,8,9]. These new techniques can identify people with osteoporosis, although they are less effective than DXA, so their use has been relegated to prescreening with subsequent DXA to confirm the diagnosis [5,21,22]. Similarly, on the basis of the hypothesis that mandibular cortical changes reflect systemic bone health, many studies have shown an association between the quality of the mandibular cortical bone and the presence of osteoporosis [10,11,12,13], making the analysis of panoramic dental radiographs a tool to help identify potential osteoporotic individuals. The best-established method for assessing bone quality by panoramic radiography is the qualitative evaluation of the MCI according to the Klemetti index [14]. In our study, lower BMD values measured by phalanx QUS and DXA in the femoral neck, trochanter, and lumbar spine were found in women with poorer mandibular cortical bone quality, as measured by the Klemetti index. These findings are consistent with the results of most studies that have investigated this association, as concluded by several reviews and meta-analyses [10,11,12,13,15]. However, there is controversy regarding whether to consider only severely eroded cortices (C3) or to include moderately eroded cortices (C2) in osteoporosis screening, as only a limited number of studies have evaluated the diagnostic accuracy of severely eroded cortices [10]. We investigated this controversy, and our results revealed that, compared with moderate cortical erosion, all BMD measures had better AUCs when severe cortical erosion was used to identify potential osteoporosis. Moreover, the femoral neck BMD had the largest AUC for severe mandibular cortical erosion.

Regarding the association between MCI and QUS, our results revealed a significant association between the Klemetti index and Ad-SOS and UBPI values, which coincides with the only published study [16] that used ultrasound to investigate this relationship. Although QUS is not suitable for diagnostic classification because the WHO criteria were developed on the basis of BMD measurement by DXA [20], it is considered a good predictor of fracture in men and women [6]. Moreover, some studies have shown that QUS is more useful than clinical risk factors for identifying women at risk for osteoporosis [24,25]. QUS can detect bone loss faster than DXA can [26], as QUS assesses bone microarchitecture. The phalanx is dominated by cortical bone with characteristics similar to those of the mandibular cortical bone; thus, alterations in the structure of one could correspond to those in the other.

The femoral neck BMD, trochanter BMD, and lumbar spine BMD, all measured by DXA in areas with a predominance of trabecular bone, were also related to MCI. However, QUS (in particular Ad-SOS and UBPI) seemed to detect moderate to severe mandibular cortical erosion better than DXA measurements, although in the case of identifying severe mandibular cortical erosion, femoral neck BMD seemed to be the best option. In addition, our predictor analysis of osteoporosis revealed that moderate (C2) and severe (C3) mandibular cortical erosion, compared with an uninjured mandibular cortical area, was independently associated with a diagnosis of osteoporosis as assessed by the WHO [20]. These findings suggest that the Klemetti index may be useful for detecting osteoporosis.

Several limitations should be considered. First, this study employed a cross-sectional design, which limits its ability to establish causality and allows only the identification of associations. Because of this, it is not possible to clarify whether changes in MCI affect BMD or vice versa. Second, all women were recruited by convenience from our region and nearby areas; thus, these results are not generalizable. The subjects of this study may be biased toward those who are highly interested in health. This is a problem regarding being representative of the population as a whole. In future studies, it will be necessary to increase the generalizability of the results by including participants from a wider range of backgrounds. Moreover, the number of participants in each group according to the Klemetti index classification was heterogeneous, so the results could be affected. Third, the mandibular cortex was evaluated using only the Klemetti index and ignoring other indices, such as the gonial index, antegonial index, panoramic mandibular index or mental index, so women with mandibular cortical erosion diagnosed by other methods may be obviated. However, the Klemetti index is the best-established method for assessing bone quality by panoramic radiography [14]. Finally, the cut-off values obtained in this study may be specific to this study population and need to be validated when applied to other populations. It would be important to validate the cut-offs in different populations in future studies.

## 5. Conclusions

In conclusion, MCI was associated with BMD measurements assessed by QUS and DXA in various bone regions. Our results suggest that the Klemetti index could be a predictor of osteoporosis and fracture risk. We do not recommend prescribing the use of a panoramic radiograph to diagnosis osteoporosis; however, when a panoramic radiograph is available, MCI could be used for prescreening to refer patients for DXA.

## Figures and Tables

**Table 1:** Biological, anthropometric, lifestyle habits, and dental parameters in this study according to the Klemetti index.

	C1 (*n* = 48)	C2 (*n* = 97)	C3 (*n* = 34)	*p*-Value
Age (years)	56.0 [51.0–60.0]	59 [55–63.5]	62.5 [58.5–67.8]	0.001 ^b,†^
Gonadal status (%)				
Premenopausal	9 (18.8%)	13 (13.4%)	1 (2.9%)	0.105 ^‡^
Postmenopausal	39 (81.3%)	84 (86.6%)	33 (97.1%)	
Menarche age (years)	12 [11–14]	13 [12–14]	12 [7.5–17.3]	0.384 ^†^
Years with menopause (years)	5 [2–10]	9 [4–13.8]	12 [7.5–17.3]	0.002 ^b,†^
Gravidity (*n*)	2 [0–3]	2 [1.5–3]	2 [0–3]	0.253 ^†^
Births (*n*)	2 [0–2]	2 [1,2]	2 [0–2.3]	0.270 ^†^
Breastfeeding (months)	3 [0–6.8]	4 [0–10]	3 [0–10]	0.410 ^†^
BMI (kg/m^2^)	25.81 ± 4.21	27.31 ± 4.92	26.87 ± 4.58	0.205 *
Obesity (%)	8 (16.7%)	25 (25.8%)	7 (20.6%)	0.447 ^‡^
WHR	0.82 ± 0.06	0.82 ± 0.07	0.81 ± 0.06	0.660 *
% Body fat	34.45 ± 6.28	35.99 ± 7.26	35.97 ± 6.71	0.438 *
Ca Supplementation (%)	8 (16.7%)	14 (14.4%)	4 (11.8%)	0.872 ^‡^
Vitamin D Supplementation (%)	9 (18.8%)	9 (9.3%)	2 (5.9%)	0.298 ^‡^
Current smoking (%)	9 (18.8%)	16 (16.5%)	6 (17.6%)	0.943 ^‡^
Physical Activity (%)				
Sedentary	15 (31.3%)	28 (28.9%)	7 (20.6%)	0.825 ^‡^
Moderate	9 (18.8%)	20 (20.6%)	9 (26.5%)	
Active	24 (50.0%)	49 (50.5%)	18 (52.9%)	
Tooth loss (*n*)	0 [0–3.8]	0 [0–5.5]	5.5 [0–10.5]	0.016 ^b,†^

Quantitative variables expressed as mean ± SD or median [interquartile ratio]. ^b^ C1 vs. C3; C2 vs. C3. * ANOVA test, ^†^ Kruskal–Wallis test; ^‡^ Chi-square test. Abbreviations: BMI: body mass index; MCW: mandibular cortical width; PMI: panoramic mandibular index; WHR: waist-to-hip ratio.

**Table 2:** Bone status and bone density according to the Klemetti index.

	C1 (*n* = 48)	C2 (*n* = 97)	C3 (*n* = 34)	*p*-Value	*p*-Value ^&^
Quantitative bone ultrasound					
Ad-SOS (m/s)	2073.52 ± 74.59	2022.41 ± 90.80	1975.43 ± 98.72	<0.001 ^a,b,c,^*	0.007 ^a,b^
UBPI	0.66 ± 0.16	0.56 ± 0.18	0.47 ± 0.17	<0.001 ^a,b,c,^*	0.005 ^a,b^
BTT (μs)	1.43 ± 0.22	1.34 ± 0.24	1.25 ± 0.29	0.007 ^b,^*	0.328
BMD (gr/cm^2^)					
BMD femur neck	0.804 ± 0.088	0.780 ± 0.110	0.714 ± 0.107	<0.001 ^b,c,^*	0.012 ^b^
BMD trochanter	0.632 ± 0.081	0.630 ± 0.100	0.578 ± 0.091	0.014 ^b,c,^*	0.048 ^b^
BMD lumbar spine (L2-L4)	0.944 ± 0.128	0.936 ± 0.179	0.850 ± 0.167	0.020 ^b,c,^*	0.027 ^b^
Osteoporosis diagnosis (%)	5 (10.4%)	24 (24.7%)	15 (44.1%)	0.002 ^a,b,c,‡^	-

^a^ C1 vs. C2; ^b^ C1 vs. C3; ^c^ C2 vs. C3. * ANOVA test; ^‡^ Chi-square test; ^&^ ANCOVA test: adjustment for age (years), gonadal status (pre- and postmenopausal), menarche age (years), years with menopause (years), BMI (kg/m^2^), WHR, % fat body, Ca supplementation, vitamin supplementation, physical activity (sedentary, moderate, and active), and smoking. Abbreviations: Ad-SOS: amplitude-dependent speed of sound; BMD: body mass density; BTT: bone transmission time; UBPI: ultrasound bone profiler index.

**Table 3:** The AUCs and optimal cut-off values of bone density for identifying mandibular moderate or severe cortical erosion.

	Moderate or Severe Cortical Erosion (Klemetti Index = C2 or C3)						Severe Cortical Erosion (Klemetti Index = C3)					
	AUC (95%IC)	*p*-Value	Sensitivity	Specificity	Youden’s Index	Cut-Off	AUC (95%IC)	*p*-Value	Sensitivity	Specificity	Youden’s Index	Cut-Off
Quantitative bone ultrasound												
Ad-SOS (m/s)	0.702 (0.618–0.786)	<0.001	0.631	0.729	1.360	<2037.5	0.703 (0.600–0.806)	<0.001	0.382	0.931	1.313	<1929.00
UBPI	0.695 (0.609–0.782)	<0.001	0.672	0.723	1.395	<0.605	0.699 (0.602–0.796)	<0.001	0.688	0.664	1.352	<0.505
BTT (μs)	0.619 (0.534–0.705)	0.016	0.416	0.872	1.288	<1.240	0.632 (0.517–0.748)	0.020	0.438	0.843	1.281	<1.160
BMD (gr/cm^2^)												
BMD femur neck	0.617 (0.531–0.704)	0.018	0.382	0.896	1.278	<0.714	0.719 (0.615–0.823)	<0.001	0.647	0.821	1.468	<0.703
BMD trochanter	0.543 (0.454–0.633)	0.373	0.313	0.896	1.209	<0.557	0.671 (0.565–0.777)	0.002	0.618	0.786	1.404	<0.566
BMD lumbar spine (L2-L4)	0.583 (0.499–0.66)	0.090	0.290	0.979	1.269	<0.779	0.677 (0.567–0.787)	0.001	0.735	0.634	1.369	<0.882

Abbreviations: Ad-SOS: amplitude-dependent speed of sound; AUC: area under curve; BMD: body mass density; BTT: bone transmission time; UBPI: ultrasound bone profiler index.

**Table 4:** Predictors of osteoporosis. Uni- and multivariate analysis.

	Univariate		Multivariate	
	OR (CI%95)	*p*-Value	aOR (CI%95)	*p*-Value
Age (1-year increase)	1.07 (1.02–1.12)	0.003	1.01 (0.93–1.08)	0.879
Postmenopausal status (%)	8.37 (1.09–64.03)	0.041	3.52 (0.33–37.53)	0.297
Menarche age (1-year increase)	1.31 (1.03–1.68)	0.028	1.29 (0.98–1.70)	0.064
Years with menopause (1-year increase)	1.05 (1.01–1.09)	0.005	1.01 (0.95–1.07)	0.665
Gravity (1-pregnancy increase)	1.09 (0.87–1.37)	0.429	-	-
Births ≥ 1 (1-birth increase)	1.15 (0.88–1.50)	0.302	-	-
Breastfeeding (1-month increase)	1.00 (0.96–1.03)	0.908	-	-
BMI ≥ 30 kg/m^2^	0.71 (0.30–1.79)	0.446	-	-
WHR ≥ 0.85	1.17 (0.58–2.38)	0.646	-	-
% Body fat (1% increase)	1.00 (0.95–1.05)	0.964	-	-
Ca supplementation (%)	3.24 (1.36–7.68)	0.008	1.73 (0.53–5.61)	0.358
Vitamin D supplementation (%)	2.89 (1.11–7.55)	0.029	3.91 (1.01–15.19)	0.049
Physical activity (%)				
Sedentary	1.00 Ref		1.00 Ref	
Moderate	2.13 (0.76–5.99)	0.148	-	-
Active	1.98 (0.82–4.81)	0.128	-	-
Current smoking (%)	0.87 (0.34–2.19)	0.776	-	-
Mandibular inferior cortex—Klemetti index				
C1	1.00 Ref		1.00 Ref	
C2	2.82 (1.01–7.95)	0.045	3.70 (1.12–12.18)	0.031
C3	6.78 (2.15–21.38)	0.001	7.88 (2.03–30.52)	0.003
Tooth loss (1-tooth loss)	1.06 (1.01–1.10)	0.006	1.03 (0.98–1.08)	0.226

Abbreviations: aOR: adjusted odds ratio; BMI: body mass index; MCW: mandibular cortical width; OR: odds ratio PMI: panoramic mandibular index; WHR: waist-to-hip ratio.

## Data Availability

Dataset available on request from the authors.
